# Risk Factors and Management Approaches for Staple Line Leaks Following Sleeve Gastrectomy: A Single-Center Retrospective Study of 402 Patients

**DOI:** 10.3390/jpm13091422

**Published:** 2023-09-21

**Authors:** Georgios-Ioannis Verras, Francesk Mulita, Charalampos Lampropoulos, Dimitrios Kehagias, Oliver Curwen, Andreas Antzoulas, Ioannis Panagiotopoulos, Vasileios Leivaditis, Ioannis Kehagias

**Affiliations:** 1 Department of Surgery, General University Hospital of Patras, 26504 Patras, Greece; 2 General Surgery, Epsom and St. Helier University Hospitals, National Health Service (NHS) Trust, London SM5 1AA, UK; 3 Department of Cardiothoracic Surgery, General Hospital of Athens “Ippokrateio”, 11527 Athens, Greece; 4 Department of Cardiothoracic and Vascular Surgery, Westpfalz-Klinikum, 67655 Kaiserslautern, Germany

**Keywords:** postoperative leak management, morbid obesity, leakage, risk factors, sleeve gastrectomy

## Abstract

Sleeve gastrectomy (SG) has gained ever-increasing popularity among laparoscopic surgeons involved in bariatric surgery. This single-institution, retrospective cohort study aims to evaluate the prevalence of postoperative staple line leakage (PSLL) after LSG and identify risk factors for its development. We included patient data that underwent LSG at our institution for a span of 17 years—starting in January 2005 and ending in December 2022. We set the investigation of correlations of patient-related factors (age, weight, BMI, smoking status, presence of diabetes mellitus) with the occurrence of postoperative leaks. A total of 402 patients were included in our study. Of them, 26 (6.46%) developed PSLL. In total, 19 (73%) patients underwent percutaneous drainage and 14 patients (53.8%) were treated with intraluminal endoscopic stenting. Finally, five patients (19.2%) were treated with endoscopic clipping of the defect. Operative management was required in only one patient. There were no statistically significant differences in patient age, mean weight at the time of operation, and mean BMI. Abnormal drain amylase levels were associated with earlier detection of PSLL. More consideration needs to be given to producing a consensus regarding the management of PSLL, prioritizing nonoperative management with the combination of percutaneous drainage and endoscopic stenting as the safest and most efficient approach.

## 1. Introduction

Laparoscopic sleeve gastrectomy (LSG) has effectively been popularized due to its effectiveness in achieving high percentages of excess weight loss (EWL), combined with its favorable safety profile mainly compared to Roux-en-Y gastric bypass (RYGB). Currently, it is the most frequently performed bariatric procedure, accounting for 58% of all such procedures worldwide [1,2,3]. The American Society for Metabolic and Bariatric Surgery (ASMBS) has recognized the significant impact of LSG, and utilizing long-term outcome data from large studies, has officially classified it as a primary bariatric operation for the management of morbid obesity [4]. Indications to undergo sleeve gastrectomy remain the same as with other types of bariatric surgery and include BMI equal or greater than 35 kg/m^2^, BMI of 30 to 35 kg/m^2^ with the presence of serious metabolic disease as a comorbidity, especially regarding poorly controlled diabetes mellitus or metabolic syndrome [5,6,7].

In addition, SG has been the operation of choice for a wide array of high-risk patients, and experts recommend it over other procedures such as RYGB. The expert consensus statement on SG states that SG is a safe and effective choice for patients awaiting transplant, the elderly, adolescents, and super obese patients [8], as well as patients requiring revision from another type of weight loss operation, mainly adjustable gastric banding [9,10,11,12,13]. Despite many years of experience and continuous reproducible results in successfully treating obesity-caused morbidity, as well as an evidently favorable safety profile, SG continues to display certain postoperative complications in approximately 2.2% of the patients [14] with reports from some centers mentioning percentages of up to 5.8% [15]. Among these, postoperative leakage is a major cause of mortality and extended hospital stays associated with LSG. It is estimated that anastomotic leakage occurs in approximately 1–3% of the patients with preoperative BMI being an effector for slightly higher rates of leakage occurrence [16]. Currently, there is an identifiable gap in the surgical literature, regarding several aspects of post-LSG leaks, evidenced by a lack of definitive guidelines for their management strategies. Outstanding issues that need to be further explored, include correlations with the timing of postoperative leaks, identification of patient risk factors associated with the development of leaks, and appropriate ranking of management options within the tertiary hospital environment.

Our retrospective, single-center study aims to evaluate the prevalence of postoperative staple line leakage after LSG and identify risk factors for its development, with a focus on acute (before the 7th postoperative day) leaks. As secondary aims of this study, we also describe patient symptomatology, the correlation of patient characteristics with a postoperative day of leakage diagnosis, and the management options of anastomotic leaks, through our practice.

## 2. Materials and Methods

For the purposes of this study, we analyzed patient data that underwent LSG at the General University Hospital of Patras for a span of 17 years—starting in January 2005 and ending in December 2022. Patient data were considered for inclusion only when the hospitalization records were complete until the patient was discharged with the ability to tolerate oral feeding, including any additional readmissions. Patients that underwent previous bariatric operations were excluded from this study. Only patients that were able to provide an informed consent form were deemed eligible for inclusion. Patients that were treated for any postoperative complication other than leakage (e.g., staple line bleeding) were not included in the study. The study population included all consecutive patients undergoing LSG that were deemed eligible by the above inclusion criteria. The present study was approved by the Ethics Committee of our institution (approval no: 11452-2/2/2023).

### 2.1. Data Procurement for Analysis

As a primary aim of our work, we set the investigation of correlations of patient-related factors (age, weight, BMI, smoking status, presence of diabetes mellitus) with the occurrence of postoperative leaks. The data extracted from patient records were used to look into the timing of postoperative leaks, in relation to patient factors and requirements for leak management, including age, weight at the time of operation, BMI at the time of operation, total hospital stay, need for nutritional support, and need for readmission. Additionally, we studied the prevalence of presenting symptoms and symptom constellations in patients with postoperative leaks. Length of initial hospital stay was defined as the number of days from the first presentation of a gastric leak, until the successful discharge of the patient, able to tolerate oral feeding. The overall time-to-resolution was calculated based on the sum of hospitalization days, over all readmissions, which was required for the complete resolution of the gastric leak.

In total, 402 patients who had undergone SG over a period of 17 years were included in our study. In addition to SG, several other types of bariatric–metabolic procedures (including RYGB and one anastomosis gastric bypass) are performed at our institution. Based on the annual number of bariatric-metabolic procedures, our institution’s bariatric center is currently considered a Level II bariatric center [17]. Preoperatively, all patients underwent a standard workup, including history, physical examination, and an extensive laboratory/imaging evaluation (blood tests, esophagogastroduodenoscopy, polysomnography, abdominal ultrasound, heart ultrasound, etc.). In addition, all patients were thoroughly evaluated by different specialties and behavioral, anthropometric parameters, and metabolic profiles were assessed. A questionnaire was completed from each participant regarding eating habits, personal medical history, and medications. All procedures were performed by bariatric consultant surgeons with more than 10 years of bariatric surgery experience. The operative technique employed by our institution remained constant, with only minor adjustments over the years that do not influence intraoperative approaches and/or outcomes.

### 2.2. Operative Technique

Anatomically, the standard SG resection begins 2 cm to 6 cm from the pylorus of the stomach. The complete mobilization of the greater curvature then follows, utilizing a vessel-sealing device, to devascularize the whole of the greater curvature, up until the diaphragmatic hiatus. With the aid of the anesthesiologist, a bougie is then inserted trans orally, that will be used to guide the resection. Current consensus states that a 36 Fr size bougie is the most widely used [17,18,19,20], with multiple bougie sizes being reported as preferred in the literature. As standard practice, we used a narrower, 32 Fr bougie for the creation of the gastric sleeve. SG resection begins 2 cm from the pylorus of the stomach for all patients. After the resection trajectory is determined, the surgeon fires the stapler, in order to achieve the “sleeving” of the stomach. Reinforcement of the staple line as a means of preventing postoperative leakage has been proposed.

Postoperative leaks were diagnosed using a multitude of diagnostic measures, including amylase levels of drains, radiographic features (UGI scans with soluble contrast and double-contrast CT scans), and clinical manifestations. A leak was suspected, based on the patient’s clinical presentation in the immediate and early postoperative period, and the serial, routine monitoring of drain amylase levels. After a clinical suspicion of postoperative leak was established, the patient underwent a double-contrast CT scan, which is known to be the most sensitive identifier of a postoperative leak. Additional testing with UGI radiographic studies was also carried out. Postoperative leaks were further categorized into three subcategories according to leak timing as: early leaks (up to 3 days postoperatively)—11 patients (42.3%), intermediate leaks (4–7 days postoperatively).

### 2.3. Statistical Analysis

**Figure 1:**
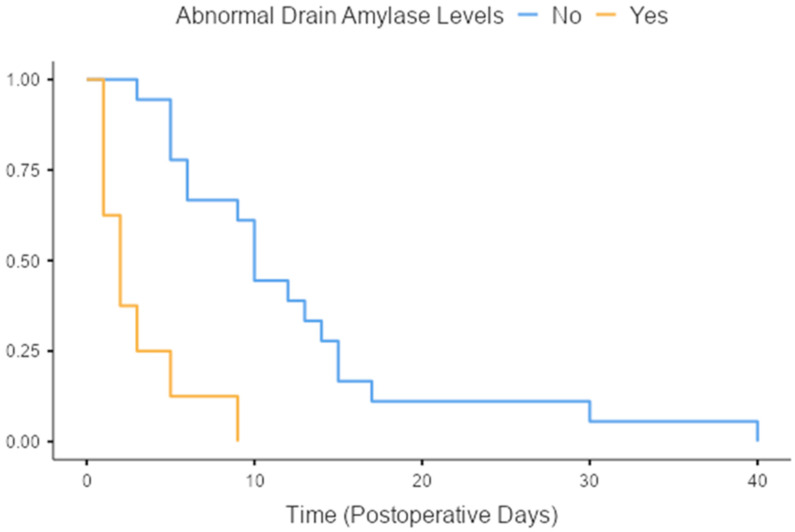
The utility of abnormal drain amylase levels as a diagnostic modality in staple line leaks.

Statistical analysis was carried out using the SPSS statistical package, as well as the R-based software Jamovi. To compare continuous variables, we used a combination of the Student’s *t*-test and the Mann–Whitney U, depending on residual distribution. Multinomial regression models were built using a combination of forward and backwards (stepwise) regression to account for confounding on the multivariable analysis. During this process, regression models were built by allowing for the addition and removal of explanatory variables, at the same time, until the model with the best data fit was selected. For the comparison of the continuous variables, Student’s t and Mann–Whitney U were used according to the normality assessment of the variable distribution. To evaluate the utility of drain amylase levels in the timing of leak diagnosis, the Cox proportional hazards regression was used to build time-to-event models for the occurrence of staple line leaks as seen in Figure 1. Time-to-event analysis was also utilized to evaluate the different treatment approaches of postoperative leaks.

## 3. Results

During the study period, data from a total of 402 patients were enrolled. Within this patient cohort, 26 patients (6.46%) developed a postoperative staple line leak. The entirety of the reported leaks were reported in the cardio-esophageal junction area. The demographic characteristics of leak vs. non-leak patients can be seen in Table 1. There were no statistically significant differences in patient age, mean weight at time of operation, and mean BMI (Table 1). Average hospital stay differed significantly between the two groups, with patients complicated by postoperative leaks, being hospitalized for a total of 44.2 (SD 27.24) days on average, as opposed to 7.78 (SD 7) days for the uncomplicated patients.

Regarding presenting symptoms of patients with postoperative leaks, developing a high or low-grade fever was the hallmark of diagnosing a postoperative leak, found in 96.2% of the patients. Other symptoms included acute abdominal pain in 38.5% of the patients, positive Kehr sign in 38.5%, respiratory distress in 19.2% of the patients, and vomiting in 15.4% of the patients (Table 2). Hemodynamic instability requiring aggressive resuscitation on presentation was found in only one (4.2%) patient of our cohort. The most common symptom constellation was that of fever with acute abdominal pain and positive Kehr sign, found in 11 (42.3%) patients. Two patients developed subclinical leaks with no reported symptoms. One was diagnosed after routine radiographical upper GI series with no change in drain biochemistry (Type B leak) and the other had solely increased amylase drain levels with no radiographic evidence of a leak (Type A leak).

Drain amylase levels area relatively controversial indicator of postoperative leakage in sleeve gastrectomy patients. The definition of abnormal amylase drain levels in our study was amylase levels higher than three times the value of patient serum amylase. In our cohort, abnormal amylase levels were detected in eight out of the twenty-six patients (30.6%) that were diagnosed with a staple line leak. Time-to-event analysis revealed that patients with abnormal amylase drain levels were diagnosed with staple line leak earlier when compared to those that had normal amylase drain levels (3 days vs. 12.5 days on average, *p* < 0.001, Figure 1).

Treatment options within our unit included conservative management with percutaneous drainage, endoscopic placement of intraluminal stents, and endoscopic clipping of the defect. In total, nineteen(73.3%) patients underwent percutaneous drainage, fourteen patients (53.8%) were treated with intraluminal endoscopic stenting, with two of them requiring a second attempt, and one requiring three attempts. Finally, five patients (19.2%) were treated with endoscopic clipping of the defect. Operative management was required in only one patient, who underwent conversion to a Roux-en-Y gastric bypass. Nutritional support was needed in 21 patients (80.8%), 11 of which received enteral feeding via NJ tube (42.3%), and 10 (38.5%) received TPN.

Readmissions due to persisting gastric leak (fistula) were deemed necessary for 15 (57.7%) patients. The number of readmissions varied from one (seven patients—26.9%) up to ten readmissions for two of our patients.

Early leaks were present in eleven patients (42.3%), intermediate leaks in four patients (15.38%), and late leaks in eleven patients (42.3%), between which subgroup analyses were conducted. Hospital stay did not differ significantly between the three subgroups in the univariate and multivariate analyses.

Choice of modality for the successful management of postoperative leaks differed significantly between the three chronological subgroups. Early leaks were successfully managed with most available modalities, drainage followed by clipping (36.36%), drainage followed by endoscopic stenting (45.45%), and conservative management (18.18%). Intermediate leaks were managed by drainage alone (50%), drainage followed by endoscopic stenting (25%), and conservatively in 25%. Late leaks were managed with drainage alone (36.36%) and drainage followed by stenting (63%) (*p* = 0.038) (Table 3).

**Figure 2:**
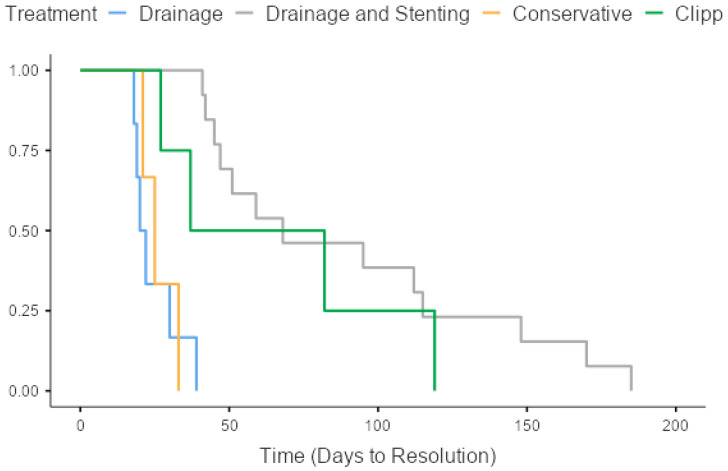
Kaplan–Meier curve for time-to-resolution stratified by treatment approach.

**Figure 3:**
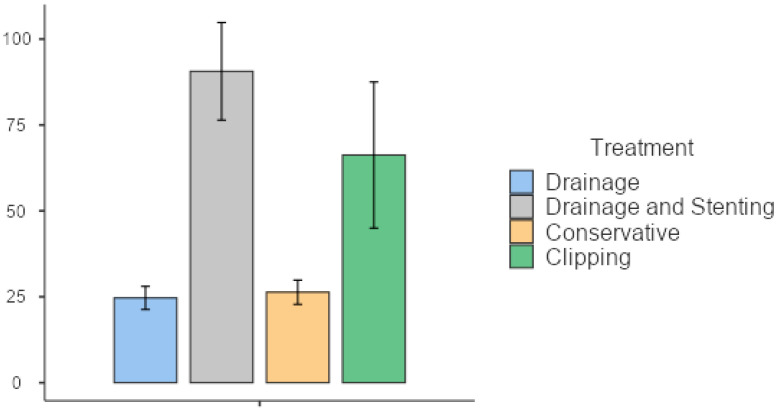
Total length of hospital stay according to management strategy.

Hospital stays differed significantly among treatment groups, with patients undergoing endoscopic clipping staying for 63.7 (SD 35.2) days, those treated with drainage and stenting being hospitalized for an average of 57.7 (SD 19.39) days, conservatively treated patients staying for 26.3 (SD 6.11) days, and those treated by drainage alone staying for 21.5 (SD 7.82) days (Table 4) (*p* = 0.008). The overall time-to-resolution was correspondingly different for the treatment groups, with drainage and stenting patients being admitted for a total of 90.6 (SD 51.2) days, endoscopic clipping patients for 66.3 (SD 42.5) days, and image-guided drainage patients for 24.7 (SD 8.24) days (*p* = 0.012) (Figure 2 and Figure 3, Table 4). Patients requiring stenting or clipping in addition to percutaneous drainage had longer hospital stays. Patients managed conservatively were not readmitted and therefore did not have a longer time-to-resolution than that of their initial hospitalization. Readmission rates also differed significantly between the three subgroups. Patients with late leaks were readmitted in 90.9% of the cases, those with intermediate leaks in 50% of the cases, followed by patients with early leaks in 27.3% of the cases (*p* = 0.001) (Table 3).

Hospital stay was also found to differ significantly when type of nutritional support was evaluated. Patients requiring no enteral or parenteral nutritional support (TPN) had an average hospitalization length of 25 days (SD 8.2), with those being supported by enteral nutrition via NJ tube having a length of stay of 44.2 (SD 22.5) days, and finally TPN-requiring patients being hospitalized for an average of 60.1 (SD 28.14) days (*p* = 0.004).

Multivariable analysis on the correlation between time-to-resolution of gastric leak, and studied parameters, revealed that neither the treatment choice, nor the need for nutritional support or the timing of gastric leak occurrence were independent predictors of hospitalization length until full resolution of the gastric leak (Table 5).

The occurrence of gastric leaks was found to be independently associated with several of the studied risk factors for leaks. Among studied patient parameters that influence the occurrence or not of gastric leaks, smoking was revealed to be an independent risk factor, with an OR of 1.43 (95 CI 1.27–1.58) (Table 6). Preoperative diagnosis of diabetes was also identified as a prognostic factor for leak occurrence with an OR of 1.13 (95 CI 1.04–1.42) as well as the presence of sleep apnea with an OR of 1.24 (95 CI 1.09–1.58). Crude preoperative BMI and BMI of more than 50 were not found to be positively related to the occurrence of postoperative leaks in our patient group (Table 6).

## 4. Discussion

Leakage is the most frequent postoperative complication of SG with occurrence rates of 2.1% on average, reaching up to 5.5% in some studies [21]. Despite the fact that SG does not involve any kind of anastomosis, it seems more susceptible to postoperative leaks than RYGB. In addition to its high prevalence rate, postoperative leakage has been found to be the second leading cause of mortality after bariatric surgery, with reported mortality rates of 1.4% [22]. No mortality that was definitively attributable to a staple line leak was recorded at our institution. Our incidence of postoperative staple line leaks is on the upper limits of that reported in the literature. One possible explanation for this difference is the fact that many of the cases included in our study are from the 2000s, when our and international experience was limited and the surgical technique was not sufficiently standardized. Another possible explanation for this difference is our election of a smaller diameter bougie tube for the creation of the gastric tube. A smaller bougie tube results in a narrower gastric pouch, which naturally produces higher intraluminal pressures that might contribute to a small increase in postoperative leaks. A few studies have looked into the correlation of bougie sizes and postoperative staple line leaks, failing to produce definitive results, yet indicating a trend towards a trade-off of increased leak incidence with better weight loss outcomes [23,24,25]. Single-institution studies are consistently producing contradictory results either in favor of or indifferent towards increased leak rates with narrower bougie sizes [25,26]. There are two meta-analyses available on the subject, with equally contradictory conclusions regarding bougie sizes and the risk of postoperative staple line leaks [27]. The analysis by Parikh et al. [27] treated bougie size as a binomial variable (greater or smaller than 40 Fr) as opposed to a continuous spectrum including all sizes which always seems to lead to no correlation between bougie size and leak. This meta-analysis managed to showcase a significant difference in postoperative leak rates for patients with bougie tubes under 40 Fr, as is also the case for our patients. Bougie size in sleeve gastrectomy is also implicated in the occurrence of chronic dehydration of the bariatric patients, with narrower gastric pouches being associated with fewer admissions for dehydration in post-SG patients [28]. At our institution, we continue to use a 32 Fr bougie. Based on our previously published results [29], we believe that a 32 Fr boogie is a key factor for successful long-term weight loss in the majority of patients. Postoperative leakage after SG is commonly classified based on time of occurrence post-surgery. According to the ASMBS, acute leaks are considered those within 7 days of surgery, early leaks are between 1 and 6 weeks postoperatively, late leaks after 6 weeks, and chronic leaks are considered those after 12 weeks [30]. Our study focused on acute and early leaks, with a few reports on late and chronic leaks that are largely thought to be governed by different pathophysiological principles. Authors have also classified leaks according to their clinical presentation, with Type I leaks being the subclinical, localized leaks without dissemination in the abdominal cavity, and Type II leaks being those with severe clinical manifestations caused by their dissemination in the peritoneal cavity [31].

The mechanics of staple line leakage, include a long stapling line being present, as well as the conversion of the stomach itself into a narrow, high-pressure tube due to the presence of both esophageal and pyloric sphincters. In our case, election of a smaller diameter bougie that results in a narrow gastric tube, also predisposes to higher intraluminal pressures that might contribute to a small increase in staple line leak rates. It must be noted however, that further morbidity rates and mortality were not increased in our cohort, compared to previous studies. Therefore, the true impact of a narrower, high-pressure tube seems to be limited in minor leaks with little to no physiological derailment of the patient. Although not leading to patient mortality, morbidity rates attributed to postoperative leaks are a significant burden for the patient and healthcare systems alike, often requiring multiple hospital admissions, as well as endoscopic and image-guided interventions. Stapler misfiring, shearing forces during surgery, and tissue creeping are also factors that contribute to early leakage, and can be combated by allowing adequate compression time before each stapler firing [31], as is our practice. As mentioned earlier, studies on staple line reinforcement do not seem to reach clear conclusions on their utility, and there while some report a trend in bleeding reduction, no clear effect on leakage rates can be inferred [32,33,34]. A meta-analysis on the subject failed to demonstrate any significant reduction in leakage rates with any of the methods studied, although postoperative bleeding and reoperation rates were significantly lower when reinforcement was applied^21^, and therefore we did not perform staple line reinforcement routinely which was abandoned post 2010, whereas negative pressure drains were placed routinely. One such study concluded that the only clear effectors on leakage rates were surgical technique, experience of the surgeon, and complexity of the patient’s anatomy [35]. In addition, our multivariate analysis determined no additional significant independent predictors for the occurrence of postoperative staple line leaks in SG patients, with the exception of smoking as a risk factor.

Experienced surgeons also seem to agree that another cause of postoperative leaks is the creation of an ischemic environment on the staple line, particularly close to and around the angle of His. Maintaining a distance of 1–2 cm from the gastro-esophageal junction, has been recommended in order to reduce tissue ischemia in this area [17,31]. Bougie size was also identified in a number of studies to be a predictor of leak occurrence [27], although as already discussed, the overall results are not definitively conclusive, largely due to the lack of homogeneity in establishing a bougie size cutoff, between the studies. In this aspect, using bougies of sizes of 40 Fr or greater seem to be associated with fewer instances of leakage [13,16,27], most likely due to the resulting wider tube that lowers the intraluminal pressure. As already mentioned, our utilization of a narrower gastric tube could be the cause behind an increased incidence rate of staple line leaks within our cohort. Patient risk factors that are found in the literature to significantly contribute to leakage rates, include male sex, BMI of more than 50 kg/m^2^, use of SG as a revision procedure, and presence of sleep apnea [36,37,38]. Interestingly, within our cohort, BMI, crude weight, gender, and ideal preoperative weight were not associated with the occurrence of staple line leaks. On the contrary, preoperative diagnosis of diabetes and sleep apnea were confirmed as independent factors contributing to leak occurrence in the multivariate analysis, which confirms previous findings from large patient cohorts [39]. This is possibly attributed to microvascular damage in diabetic patients leading to micro ischemic damage along the staple line, causing leakages to occur. Obstructive sleep apnea is also associated with increased odds for postoperative staple line leaks with two physiological mechanisms; anemia and poor tissue oxygenation are both implicated in postoperative leaks [40,41]. Revision procedures were not included in our patient cohort.

Gastric leakage can be the cause of significant mortality and morbidity of patients, leading to sepsis, hemodynamic instability, and MODS. Clinical presentation of this complication can vary from asymptomatic (Type I), to mild pain, to full-blown presentation of peritonitis with septic shock (Type II). Tachycardia, sudden postoperative abdominal pain, and fever should alert the clinician to the possibility of a developing leak [42,43,44]. Postoperative pain and fever were the most common symptom combination within our cohort, and fever developed in 96% of the patients, indicating a series of clinically significant leaks. There were no asymptomatic postoperative leaks within our cohort of patients, which raises the question of the utility of classification according to presentation.

High-quality data from RCTs have shown no statistically significant difference in anastomotic dehiscence in patients with and without intra-abdominal drains, as the current trend recommends the use of intra-abdominal drains only in complicated or revisional cases. Our study showed that the use of drains can provide early identification of PSLLs after SG, and at our institution, we continue to use them routinely. In uncomplicated cases, the intra-abdominal drain is removed on the 4th postoperative day, and only if there are alarming clinical and laboratory findings for PSLL, it remains for a longer period. We believe that early detection of a PSLL is important and may modify clinical decision-making, although we cannot be certain whether such knowledge affects patient prognosis and outcome.

Treating the postoperative leaks after SG remains a field lacking a standard algorithm, despite many surgical teams having developed several individual ones. While timely diagnosis and treatment is considered ideal and contributes to postoperative outcomes, still most patients are diagnosed with gastric leaks after they have been discharged from the hospital [22,31]. These patients contribute to late and chronic leak rates, and are the cause behind more advanced diagnosis in postoperative days. Patients presenting acutely, with fulminant sepsis and hemodynamic instability should be taken to the operating room immediately for emergency surgical intervention, rather than using any of the conservative or endoscopic methods of treatment. Managing source control of the sepsis via peritoneal lavage and drainage, is of the utmost importance in such cases, and should not be delayed in order to attempt conservative management [30,42,44]. Patients that ultimately undergo emergency surgery as a measure of leakage treatment, ultimately exhibit larger mortality rates; however, it must be noted that these are the most critically ill patients, and none of the studies measuring mortality rates have done so while adjusting for well-known mortality factors, such as organ dysfunction, presence of shock, etc. [44]. Fortunately, such cases are scarce, and in our cohort that represents a single-center experience over multiple years, there was only one such case. When considering patients without such a catastrophic presentation, a variety of management options have been described in the literature. Authors advocate attempting nonoperative management in stable patients whether the leak is early or late. Administration of broad-spectrum antibiotics for coverage of intra-abdominal infections, as well as nutrition and hydration support, are key in the conservative management of such patients [45,46,47,48]. Within the minimally invasive spectrum of treatments, percutaneous drainage of any recognized and accessible fluid collection should also be performed. In our study, percutaneous drainage with or without intraluminal stenting was the most commonly followed management strategy and resulted in significantly faster leak resolution rates and shorter hospitalization length.

Utilization of endoscopic methods in the treatment of SG leaks is also widely described in the literature. Endoluminal stenting in select patients can allow for nonoperative management of leaks, without the need for surgery, while at the same time the patients maintain nutritional support of themselves with oral feeding. Patients treated with endoluminal stents achieve high success rates of up to 100% in several studies [49,50,51,52,53], including our own. Endoscopic stenting was used in 53.8% of our patients with a perfect success rate. It must be noted however, that endoscopic stenting required additional attempts in a few of our patients, and thus, resulted in an increased average hospitalization length. Additionally, endoscopic stenting required a longer period until the confirmation of biochemical and clinical resolution of the staple line leak. Instead of primary closure (that is not recommended), covered stents manage to endoluminally seal the leaks, acting as a physical barrier that promotes healing. Shortcomings of this approach include migration of covered stents that is reported in 17–58% of the patients, requiring repetition of the endoscopy procedure [54,55]. Partially covered stents have been used to achieve better anchoring; however, in 9% of the patients, removal is unsuccessful and can require further manipulation [54,55,56,57,58]. Endoscopic clip placement can also be used to approximate the defect causing the leak and was utilized in five of our patients with no reported adverse events. This is a more technically demanding approach and requires experienced endoscopists in order to achieve good visualization and complete closure of the defect. Despite lacking large-scale trials, the literature suggests high success rates with minimal to no adverse effects [50,59,60]. In the event of failure of effective management with any of the aforementioned methods, surgical management should be the next logical step, since the time interval will likely be such, that the leak will now be considered chronic [61]. Revision options include fistulojejunostomy, RYGB conversion, and total or near total gastrectomy, or the described rendezvous procedure, a technically challenging undertaking that has yet to prove palpable advantages [62,63,64,65].

In light of a postoperative complication that can cause a great morbidity and mortality burden, to bariatric patients, novel and innovative approaches to address the postoperative leak are continuously emerging. Perhaps one of the most commonly discussed is the use of biological glue (commonly fibrin or cyanoacrylate). Fibrin glue has been studied in the closure of small leaking defects in various bariatric operations including sleeve gastrectomy. Reported success rates range from 92.8% up to 100%; however, these are reported usually in small cohorts, and after multiple application sessions. Fibrin glue increases the cellular response to damage and promotes fibroblast proliferation. Ease of application and lack of debilitating complications are the main advantages of the technique [63,64,65]. A group of researchers successfully utilized mesenchymal stem cells derived from each patient to endoscopically assist in sealing identified orifices after SG by submucosal injection with no reported complications. Open-pore suction devices can be placed endoscopically at the endoluminal fistula opening [66]. Addition of laparoscopic assistance forms the rendezvous procedure, during which the laparoscopic surgeon assists in accurate identification of the defect and placement of the suction device which is guided at the same time by the endoscopist [67]. This procedure is thought to be reserved for treatment-resistant leaks, or large leaks requiring laparoscopic washout in addition to attempts for sealing. Advanced surgical options after conservative, endoscopic, or minimally invasive approaches have failed are also being performed in experienced bariatric centers. One such approach is the complex laparoscopic Roux-en-Y fistulojejunostomy formation usually performed for complex, chronic fistulas after a SG leak [67].

### Study Limitations

Our study is not immune to certain shortcomings, as with any original report. First of all, there is an inherent difficulty in the generalization and external validity of our findings, since our study is a retrospective, single-institution cohort study. As such, our patient population was somewhat limited in a specific geographical area, and intraoperative techniques limited to those of our institution. Second, our study did not include certain proposed management strategies of staple line leaks (e.g., biological glue), due to lack of availability. Third, the retrospective nature is also a setback, since without randomization, our results while significant, are of lower quality than an RCT looking into the comparison of different management strategies for staple line leaks. Fourth, our study includes patients who underwent SG during a long time period of 17 years. This implies a lower level of experience during the first years that we performed the surgery. In addition, the indications and decisions for the various therapeutic interventions for PSLLs changed slightly or more over time. Fifth, the subgroup statistical analysis included subgroups with a small number of patients, which may not be representative. Finally, given the retrospective nature of this study design, there were several parameters that could not be evaluated and were related to the patients (e.g., clinical and laboratory findings at the time of PSLL), to PSLL (e.g., location, size, presence of abscess or fistula), and to the therapeutic methods applied (e.g., availability, indications).

## 5. Conclusions

Postoperative staple line leaks after sleeve gastrectomy, while rare, remain a potentially catastrophic complication. Previously reported patient risk factors were not confirmed as independent predictors, with the exception of smoking. We found that conservative management of staple line leak patients that includes percutaneous drainage and endoscopic stenting, was characterized by high success rates, low readmission rates, and shorter time-to-resolution of the leak. Abnormal drain amylase levels were associated with earlier detection of staple line leaks, and are potentially a valuable asset in the diagnostic armamentarium of the bariatric surgeon. More consideration needs to be given to producing a consensus regarding the management of staple line leaks after SG, prioritizing nonoperative management with the combination of percutaneous drainage and endoscopic stenting as the safest and most efficient approach.

## Figures and Tables

**Table 1:** Demographic characteristics of patients (as mean and SD).

	Leak Group	No Leak Group	*p*-Value *
Age	34.8 (9.8)	35.42 (9.85)	0.741
Height	170.8 (9.31)	169.04 (9.07)	0.313
Ideal Weight	64.4 (7.93)	63.59 (7.72)	0.715
Weight at Time of Operation	130.2 (16.34)	127.28 (19.88)	0.279
BMI at Time of Operation	44.7 (2.83)	44.38 (4.15)	0.249
Hospital Stay	44.2 (27.24)	7.78 (1.44)	<0.001

* calculated using Mann–Whitney U or Student’s *t*-test.

**Table 2:** Presenting symptoms of patients with gastric leaks.

Patient Presenting Symptom	Number of Reporting Patients (%)
Fever	25 (96.2%)
Respiratory Distress	5 (19.2%)
Acute Abdominal Pain	10 (38.5%)
Kehr Sign	11 (42.3%)
Vomiting	4 (15.4%)
Hemodynamic Instability	1 (4.2%)
Macroscopic Change in Drain Output	5 (19.2%)
Abnormal Amylase Drain levels	8 (30.8%)
Fever + Acute Abdominal Pain + Kehr Sign	11 (42.3%)

**Table 3:** Subgroup Analysis.

	Leak Classification (Chronological)		
Treatment	Late	Early	Intermediate
Drainage	4	0	2
Drainage and Stenting	7	5	1
Conservative	0	2	1
Clipping	0	4	0
Readmission			
Not Readmitted	1	8	2
Readmitted	10	3	2

**Table 4:** Average days of hospital stay and overall time-to-resolution per treatment method.

Treatment	Total Time to Gastric Leak Resolution (SD)	Hospital Stay (SD)
Drainage	24.7 (8.24)	21.5 (7.82)
Drainage and Stenting	90.6 (51.2)	57.7 (18.7)
Conservative	26.3 (6.11)	26.3 (6.11)
Clipping	66.3 (42.5)	63.5 (38.1)

**Table 5:** Days to Resolution—Multivariate Analysis.

Predictor	Beta Coefficient	SE	*t*	*p*
Intercept ᵃ	−0.0593	114.795	−5.16 × 10^−4^	1.000
Leak Classification (Chronological):				
Late–Early	8.2717	21.180	0.3905	0.702
Intermediate–Early	13.0998	20.938	0.6256	0.542
BMI at Time of Operation	−0.0452	2.558	−0.0177	0.986
Age	0.2198	0.506	0.4341	0.671
Weight at Time of Operation	−0.0998	0.462	−0.2158	0.832
Treatment:				
Drainage and Stenting–Drainage	34.6972	13.599	2.5515	0.023
Conservative–Drainage	10.2674	20.442	0.5023	0.623
Clipping–Drainage	34.9074	24.583	1.4200	0.177
Nutritional Support:				
Enteral Nutrition (NJ Tube)–Normal Enteral Nutrition	9.0417	23.072	0.3919	0.701
Total Parenteral Nutrition–Normal Enteral Nutrition	24.4553	20.511	1.1923	0.253
BMI 50:				
Less than 50–More than 50	11.5484	28.366	0.4071	0.690

ᵃ Represents reference level.

**Table 6:** Gastric Leak Occurrence—Multivariate Analysis.

Predictor	Odds Ratio	*p*-Value
Age	1.006	0.737
Weight at Time of Operation	0.990	0.456
BMI at Time of Operation	1.019	0.767
BMI More than 50	0.5059	0.496
Smoking	1.09	0.023
Diabetes	1.13	0.034
Sleep Apnea	1.24	0.041

## Data Availability

Study data are available after request to the authors and appropriate clearance from the Bioethics Committee of the General University Hospital of Patras.
